# Scaling-up Transformation of Multisensor Images with Multiple Resolutions

**DOI:** 10.3390/s90301370

**Published:** 2009-02-26

**Authors:** Shaohui Chen, Renhua Zhang, Hongbo Su, Jing Tian, Jun Xia

**Affiliations:** Key Laboratory of Water Cycle and Related Land Surface Processes, Institute of Geographic Sciences and Natural Resources Research, Chinese Academy of Sciences, 11A, Datun Road, Chaoyang District, Beijing 100101, P.R. China

**Keywords:** Intensity Hue Saturation, Generalized Intensity Modulation, Spectral Response Function, Empirical Mode Decomposition, Scaling-up Transformation

## Abstract

For scaling up low resolution multispectral images (LRMIs) with high resolution panchromatic image (HRPI), intensity-hue-saturation (IHS) can produce satisfactory spatial enhancement but usually introduces spectral distortion in the fused high resolution multispectral images (HRMIs). In this paper, to minimize this problem, we present a generalized intensity modulation (GIM) by extending the IHS transform to an arbitrary number of LRMIs, which uses the information of the spectral response functions (SRFs) of the multispectral and panchromatic sensors. Before modulation, the generalized intensity is enhanced by injecting details extracted from the HRPI by means of empirical mode decomposition. After the enhanced generalized intensity is substituted for the old one, the HRMIs are obtained through the GIM. Quickbird images are used to illustrate the superiority of this proposed method. Extensive comparison results based on visual analysis and Wald’s protocol demonstrate that the proposed method is more encouraging for scaling up the LRMIs with the HRPI spectrally and spatially than the tested fusion methods.

## 1. Introduction

In many remote sensing applications that require both high spatial and high spectral resolution, such as urban mapping, vegetation identification and land use classification, high resolution panchromatic images (HRPIs) and low resolution multispectral images (LRMIs) are fused using fusion methods to produce high resolution multispectral images (HRMIs), not only to increase the ability of humans to interpret the image dataset, but also for improving the accuracy of the classification [1].

Many image fusion methods have been proposed [1–3]. Initial methods mainly focused on intensity modulation for sharpening the LRMI by means of an HRPI. These methods provide good visual HRMIs, while overlooking the requirement of the high quality synthesis of spectral content which is very important for most remote sensing applications based on spectral signatures, such as soil and lithology [4]. Another family of methods, such as high pass filtering (HPF) [5] and gradient pyramid [6], yields HRMIs with much less spectral distortion by injecting high frequency information from the HRPI into the LRMI. However, it is not until the introduction of methods based on multiresolution analysis that HRMI achieved artistic results [7]. Conventional image fusion approaches based on à trous wavelet transform (AWT) [8] implement multiresoltuion decomposition on the HRPI, and then the HRMI can be recovered by performing the inverse AWT (IAWT) from the LRMI and the wavelet planes of the HRPI. However, wavelet based fusion methods do not consider the differences in high frequency information between the HRPI and the LRMIs [9].

The Intensity Hue Saturation (IHS) method can quickly merge massive volumes of data by requiring only resampled LRMIs aside from its high spatial enhancement capability [10]. Its concept is based on the representation of the LRMIs in the IHS system, and then substituting the low resolution intensity component (LRIC) with the HRPI. The inverse IHS transformation allows one to produce the HRMIs. However, the use of such a method for multisensor image fusion often leads to important modifications of the spectral properties of the LRMIs. This is due to the fact that all details contained in the HRPI are directly substituted to the LRIC [10].

A more appropriate use of the IHS method should rather consist of fusing the LRIC with the HRPI through image processing techniques to produce one high resolution intensity component (HRIC). For this purpose, empirical mode decomposition (EMD) is introduced into the fusion of the LRIC with the HRPI. The EMD is a recent method for analyzing nonlinear and nonstationary data, developed by Huang *et al*. [11]. The final representations of the signal are finite intrinsic mode functions (IMFs) that give not only sharp identifications of salient information but also smooth part of the signal. By manipulating the IMFs, the EMD is very suitable for image fusion [12].

This paper presents a novel scaling up multisensor image fusion method, based on the joint use of generalized intensity modulation (GIM) and the EMD. The GIM is the generalization of the IHS transform, and it incorporates information from the spectral response functions (SRFs) of the LRMI and the HRPI sensors to estimate the LRIC. The EMD is used to extract the spatial details of the HPRI to be injected into the LRIC. As a result, one texture modulated HRIC is produced. Experimental results based on Quickbird images are presented and discussed. Visual analysis and quantitative comparison demonstrate that the new approach provides a satisfactory result, both visually and quantitatively.

## 2. Combined GIM-EMD Image Fusion Method

### 2.1. GIM based fusion method

The main advantage of the IHS method lies in the separation of spatial information such as an intensity (*I*) component from the spectral information represented by the hue (*H*) and saturation (*S*) components. One can independently manipulate the *I* component while maintaining the overall color balance of the original images. Traditionally, the IHS method comprises four steps: 1) transform three LRMIs to IHS components; 2) match the histogram of the HRPI with that of the LRIC; 3) replace the LRIC with the stretched HRPI; and 4) inverse-transform IHS channels to three HRMIs.

(1) $$\begin{array}{cc} \text{Forward transform}: & \left[\begin{array}{c} \mathrm{LRIC}\\ H\\ S \end{array}\right]=\left[\begin{array}{ccc} \frac{1}{3} & \frac{1}{3} & \frac{1}{3}\\ \frac{-1}{\sqrt{6}} & \frac{-1}{\sqrt{6}} & \frac{2}{\sqrt{6}}\\ \frac{1}{\sqrt{6}} & \frac{-1}{\sqrt{6}} & 0 \end{array}\right]\;\left[\begin{array}{c} {\mathrm{LRMI}}_{1}\\ {\mathrm{LRMI}}_{2}\\ {\mathrm{LRMI}}_{3} \end{array}\right] \end{array}$$

(2) $$\begin{array}{cc} \text{Backward transform}: & \left[\begin{array}{c} {\mathrm{HRMI}}_{1}\\ {\mathrm{HRMI}}_{2}\\ {\mathrm{HRMI}}_{3} \end{array}\right]=\left[\begin{array}{ccc} 1 & \frac{-1}{\sqrt{6}} & \frac{3}{\sqrt{6}}\\ 1 & \frac{-1}{\sqrt{6}} & \frac{-3}{\sqrt{6}}\\ 1 & \frac{2}{\sqrt{6}} & 0 \end{array}\right]\;\left[\begin{array}{c} \mathrm{HRPI}\\ H\\ S \end{array}\right]=\left[\begin{array}{c} {\mathrm{LRMI}}_{1}+(\mathrm{HRPI}-\mathrm{LRIC})\\ {\mathrm{LRMI}}_{2}+(\mathrm{HRPI}-\mathrm{LRIC})\\ {\mathrm{LRMI}}_{3}+(\mathrm{HRPI}-\mathrm{LRIC}) \end{array}\right] \end{array}$$

Inspired by (2), a GIM method for one to *N* fusion can be deduced as:

(3) $$\begin{array}{cc} \text{Forward transform}: & \mathrm{LRIC}=\frac{\sum_{n=1}^{N}\alpha_{n}{\mathrm{LRMI}}_{n}}{N} \end{array}$$

(4) $$\begin{array}{cc} \text{Backward transform}: & \left[\begin{array}{c} {\mathrm{HRMI}}_{1}\\ \vdots \\ {\mathrm{HRMI}}_{N} \end{array}\right]=\left[\begin{array}{c} {\mathrm{LRMI}}_{1}+(\mathrm{HRPI}-\mathrm{LRIC})\\ \vdots \\ {\mathrm{LRMI}}_{N}+(\mathrm{HRPI}-\mathrm{LRIC}) \end{array}\right] \end{array}$$

In (3), α*_n_* is the weight coefficient of the LRMI*_n_*, which is related with the SRFs of the *n*th multispectral and panchromatic sensors, and is discussed in the following section.

### 2.2. Production of the LRIC based on SRF

The SRF of a sensor defines the probability that the radiation is detected by this sensor. For producing the LRIC from the {LRMI*_n_*}_1≤*n*≤*N*_ and the HRPI, the SRF of the panchromatic sensor (*φ*(*υ*)) and the SRFs of the *N* multispectral sensors ({*ψ_n_*(*υ*)}_1≤*n*≤*N*_) are involved. Let the events *m_n_* and *t* be the detection of the radiation by the *n*th multispectral sensor and the HRPI sensor, respectively. The probabilities of the events *m_n_* and *t* are [7]:

(5) $$P(m_{n})=\int \psi_{n}(\upsilon )d\upsilon$$

(6) P(t)=∫φ(υ)dυ

The probability of the radiation detected by both sensors (event *m_n_*∩*t*) is:

(7) $$P(m_{n}\cap t)=\int \text{min}(\varphi (\upsilon ),\psi_{n}(\upsilon ))d\upsilon$$

**Figure 1.**
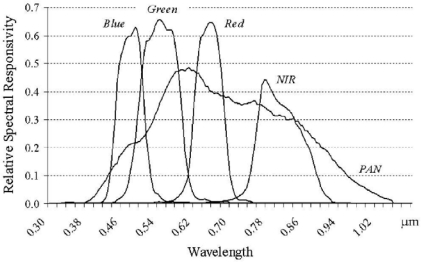
Spectral response functions for QuickBird-2 bands.

In geometrical terms, *P*(*m_n_*∩*t*) can be understood as the area below *φ*(*υ*) and *ψ_n_*(*υ*) (Figure 1, http://www.spaceimaging.com/producs/QuickBird/QuickBirdRelativeSpectralResponse.xls, accessed on July, 8, 2005).

Given the radiation detected by the *n*th multispectral sensor, the probability to be detected by the HRPI sensor is:

(8) $$P(t|m_{n})=\frac{P(m_{n}\cap t)}{P(m_{n})}$$

From (8), we can obtain a new LRIC as:

(9) $$\mathrm{LRIC}=\sum_{\forall n}\alpha_{n}{\mathrm{LRMI}}_{n}$$

(10) $$\alpha_{n}=\frac{P(t|m_{n})}{\sum_{\forall n}P(t|m_{n})}$$

where *α_n_* is the spectral signature contribution factor of the LRMI*_n_* to the LRIC, and preserves the spectral properties of the scanned objects when producing the LRIC. That is, *α_n_* is the ratio of the spectral content identified by the HRPI sensor from what the LRMI*_n_* records to that identified by the HRPI sensor from all LRMI bands.

### 2.3. Introduction of EMD into the fusion of the LRIC and the HRPI

The IHS method for multisensor image fusion often causes significant spectral distortion in the HRMIs. This is due to the fact that all details contained in the HRPI are directly substituted to the LRIC [10]. A more suitable use of the IHS method should rather fuse the LRIC with the HRPI through an advanced image processing technique to produce a better HRIC. The EMD is a highly efficient and adaptive algorithm for analyzing nonlinear and nonstationary signal [11]. With the development of the EMD, one expects much room for improvement over the simple substitution scheme.

The EMD can decompose a signal into finite intrinsic mode functions (IMFs) and one residue component. Each IMF represents simple oscillatory mode imbedded in the signal [11]. Hence, the EMD offers higher frequency resolution and more accurate timing of nonlinear and nonstationary signal events than traditional integral transforms, and the sum of all IMFs match the original signal perfectly using the inverse EMD (IEMD). For the basic theory of the EMD, interested readers may consult [11] for more details.

For a two dimensional image, the sifting procedure of the EMD is summarized as follows:

1. Treating the original image *I* as the initial residue component *I*_0_.
2. Finding all the local *extrema*, then constructing two smooth cubic splines connecting all the local *maxima* and *minima* along rows to get upper envelope *u*_r_ and lower envelope *l*_r_. Similarly, upper envelope *u*_c_ and lower envelope *l*_c_ along columns are also obtained. The mean plane *ul* is defined:
  (11) $$\mathrm{ul}=\left(u_{r}+l_{r}+u_{c}+l_{c}\right)/4$$
  Then, the difference between *I*_0_ and *ul* is:
  (12) $$\omega_{1}=\;I_{0}-\mathrm{ul}$$
  This is one iteration of obtaining the IMF. Checking whether or not *ω*_1_ is an IMF: if not, treating *ω*_1_ as *I*_0_, and go to 2); if *ω*_1_ is an IMF, and treating the following residue component as *I*_0_ and go to 2):
  (13) $$I_{1}=\;I_{0}-\omega_{1}$$
  Because the value of *ul* decreases rapidly for the first several iterations and then decreases slowly, this suggests that the number of iterations can be used as the stopping criterion. Therefore, the appropriate number of iterations to obtain the IMF is used as the stopping criterion.
3. Treating the residue component as the new input. A series of {*ω_j_*}_1≤_*_j_*_≤_*_J_* is obtained by repeating 2) until *I_J_* is a monotonic component (*J* denotes the decomposition level). *I* can be recovered using the IEMD:
  (14) $$I=\;\sum_{j=1}^{J}\omega_{j}+I_{J}$$

**Figure 2.**
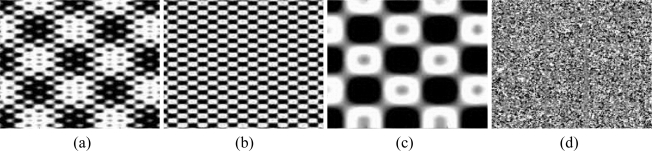
(a) The original image; (b) IMF1; (c) IMF2; (d) the residue component.

Figure 2 shows one example of the EMD. The original image was downloaded from http://www.inrialpes.fr/is2/people/pgoncalv (accessed in April 2007). Before and after the EMD, it is interesting to find that the original image contains three kinds of patterns, and the two modes and the residue component provide very useful information on a series of pattern structures which vary in scale from the smallest to the largest. Hence, the framework of the EMD is suitable for fusing multisensor images by managing the IMFs.

### 2.4. Combined GIM-EMD scaling-up transformation method

**Figure 3.**
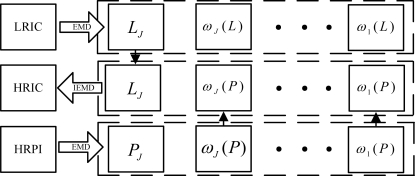
Schematic flowchart of the fusion of the LRIC and the HRPI

The fusion of the LRIC and the HRPI based on the EMD can be considered as constructing one HRIC with the same spectral response as the LRIC and the same spatial response as the HRPI. With the EMD, we expect much room for improvement over the traditional IHS fuser. The proposed procedure takes the following steps (Figure 3):

1. Obtaining the LRIC using formula (3).
2. Matching the histogram of the HRPI to that of the LRIC.
3. Decomposing the HRPI with the EMD to *J* levels, resulting in one residue component (*P_J_*) and a total of *J* detail subbands ({*ω_j_*(*P*)}_1≤*j*≤*J*_). Decomposing the LRIC with the EMD to *J* levels, resulting in a residue component (*L_J_*) and a total of *J* IMF planes ({*ω_j_*(*L*)}_1≤*j*≤*J*_).
4. Synthesizing the HRIC using *L_J_* and the *J* detail subbands ({*ω_j_*(*P*)}_1≤*j*≤*J*_) of the HRPI as:
  (15) $$\text{HRIC}=L_{J}+\sum_{j=1}^{J}\omega_{j}(P)$$
5. Replacing the LRIC with the HRIC, and obtaining *N* HRMIs as:
  (16) $${\text{HRMI}}_{n}={\text{LRMI}}_{n}+\text{HRIC}-\text{LRIC}$$

## 3. Experiments

**Figure 4.**
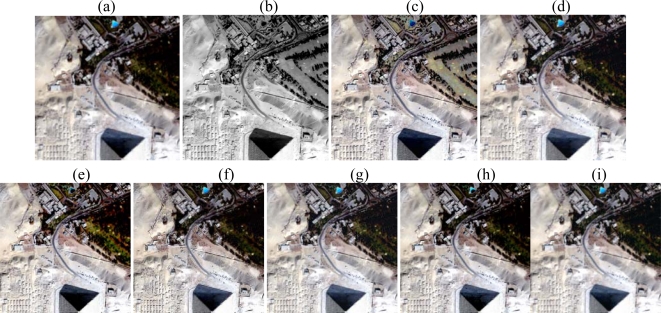
(a) the original LRMIs; (b) the HRPI; (c)–(i) the HRMIs from the IHS, AWT, BT, DWT, HPF, HPM, and the proposed method, respectively.

The raw images were downloaded from http://studio.gge.unb.ca/UNB/images. These QuickBird images cover over the Pyramids area of Egypt and were taken in 2002. The test images of size 1024 by 1024 at the resolution of 0.7 m are cut from the raw images. The panchromatic band (450–900 nm) of 0.7 m resolution and blue (450–520 nm), green (520–600 nm), red (630–690 nm), near infrared (760–900 nm) bands of 2.8 m resolution are used as the HRPI and LRMIs, respectively. Figure 4(a) displays the LRMIs in color image by mapping the red, green, blue bands into the RGB color space. Figure 4(b) shows the HRPI. Before the image fusion, the LRMIs were co-registered to the HRPI.

**Figure 5.**
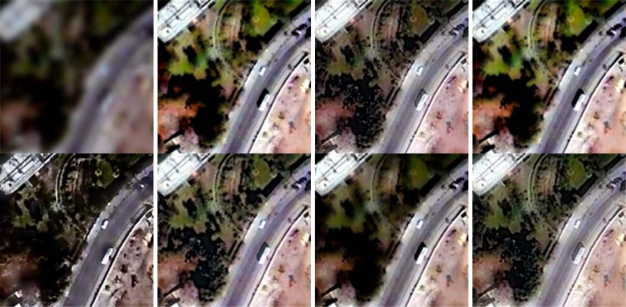
Subscenes of the original LRMIs and the HRMIs produced by different methods. (Left to right sequence) Original LRMIs, IHS, AWT, BT, DWT, HPF, HPM, and the proposed method.

For comparison purposes, the IHS, AWT, Brovey Transform (BT), Dyadic Wavelet Transform (DWT), HPF, High Pass Modulation (HPM) based fusion methods were also done. Figures 4(c)–(i) shows the HRMIs of fusing Figure 4(a) with Figure 4(b) by the seven methods. For better evaluation, Figure 5 shows subscenes of size 200×200 from the LRMIs and the corresponding HRMIs.

The qualities of the HRMIs are estimated both qualitatively and quantitatively. Visual inspection is used for qualitative estimation since visual inspection is an effective tool for analyzing local as well as global variations of spatial structures and spectral information of the HRMIs. Wald’s protocol is used to assess the qualities of the HRMIs quantitatively.

### 3.1. Visual inspection

Visual inspection provides an overall impression of image clarity and the similarity of the original and fused images. Visual analysis shows that the spatial resolution of the HRMIs is much higher than that of the LRMIs. The HRMIs present more details without noticeable spectral distortion except that of the IHS method, such as edges and slopes. Many textures and details in the HRMIs, such as edges and lines, can be identified individually in each of the HRMIs. This means that all of the fusion methods can improve the spatial quality of the LRMIs via the fusion procedure.

From Figures 4(c)–(i), it can be found that the HRMIs [Figures 4(c) and 4(e)] produced by the IHS and BT methods show obvious spectral distortion; the HRMIs [Figures 4(d) and (f)–(h)] produced by the AWT, DWT, HPF, and HPM methods show second color distortion; the HRMIs [Figure 4(i)] produced by the proposed method show the least spectral distortion. It can be concluded from Figure 4 that the HRMIs [Figure 4(i)] produced by the proposed method appear the best among the HRMIs, and the integration of spatial features and color is natural. This effect can be seen clearly in Figure 5 by enlarging a region of interest. For the IHS and BT methods, this is due to the fact that all details contained in the HRPI are directly injected into the LRMIs [10]. For additive methods, such as AWT, HPF, and HPM, this is probably due to over enhancement along the edge area because these methods have not considered the differences in high frequency information between the HRPI and the LRMIs [4]. For the DWT method, the critically sampled multiresolution analysis does not preserve the translation invariance [3].

### 3.2. Quantitative comparison

In addition to visual analysis, the performance of each method is further quantitatively analyzed by checking Wald’s protocol [13] using the following quantitative indexes.

1. Correlation coefficient (CC) between each band of the original LRMIs and the HRMIs.
2. Root mean square error (RMSE) between the LRMI and the HRMI, computed using the following equation:
  (17) $${\text{RMSE}}^{2}={\text{bias}}^{2}+{\text{SDD}}^{2}$$
  where the bias is the difference between the mean values of the LRMI and the HRMI and SDD the standard deviation of the difference image. RMSE should be as close to 0 as possible.
3. Spectral angle mapper (SAM) is defined as:
  (18) $$\text{SAM}=\text{arccos}\left(\frac{\sum_{i}u_{i}v_{i}}{\sqrt{\sum_{i}u_{i}^{2}\sqrt{\sum_{i}v_{i}^{2}}}}\right)$$
  where {*u_i_*} and {*v_i_*} denote the spectral vectors of images *U* and *V*, respectively. It should be as close to 0 as possible.
4. Relative average spectral error (RASE) characterizes the average performance of image fusion method in the spectral bands considered [13]:
  (19) $$\text{RASE}=\frac{100}{M}\sqrt{\frac{1}{N}\sum_{i=1}^{N}{\text{RMSE}}^{2}(B_{i})}$$
  where *M* is the mean radiance of the *N* LRMI bands (*B_i_*). RASE should be as close to 0 as possible.
5. Q_4_, defined as [14]:
  (20) $$Q_{4}=\frac{4[E[x\cdot y^{*}]-\bar{x}\cdot {\bar{y}}^{*}]}{E[{||\bar{x}||}^{2}]-{||\bar{x}||}^{2}+E[{||\bar{y}||}^{2}]-{||\bar{y}||}^{2}}\cdot \frac{||\bar{x}||\cdot ||\bar{y}||}{{||\bar{x}||}^{2}+{||\bar{y}||}^{2}}$$
  where *x* and *y*, which denote the four band LRMIs and the HRMIs, respectively, are both expressed as quaternions (e. g. *x*=*x*_1_+*i·x*_2_+*j·x*_3_+*k·x*_4_). *E*[·] denotes the expected value, *x̄* is the quaternion obtained by averaging the four LRMIs, and ||*x*|| is the magnitude of the quaternion. It should be as close to 1 as possible.
6. Erreur relative globale adimensionnelle de synthèse (ERGAS) [13] is given by:
  (21) $$\text{ERGAS}=100\frac{h}{l}\sqrt{\frac{1}{N}\sum_{i=1}^{N}\frac{{\text{bias}}_{i}^{2}+{\text{SDD}}_{i}^{2}}{M_{i}^{2}}}$$
  where *h* is the resolution of the HRPI, *l* the resolution of the LRMI, *N* the number of HRMIs, and *M_i_* the mean of the HRMI*_i_*. Bias is the difference between the mean of the LRMI and HRMI, and SDD the square root of the difference image between each band of the LRMIs and the HRMIs.

Three criteria based on the Wald’s protocol were employed to test the degree of spectral distortion caused by the fusion methods [14]: (1) In order to test the first property of Wald’s protocol, the HRMIs are spatially degraded to the resolution level of the original LRMIs (2.8 m) by cubic interpolation. Then, the degraded HRMIs (DHRMIs) are compared with the original LRMIs. Table 1 shows the results. (2) In order to test the second and third properties of Wald’s protocol, the fusion results (LHRMIs) of the degraded HRPI and LRMIs (4 times degraded in resolution by cubic convolution) are also compared with the LRMIs. Table 2 shows the results. In Tables 1 and 2, B_1_, B_2_, B_3_ and B_4_ denote the red, green, blue, and near infrared bands, respectively, and the last column reflects the ideal situation that should be reached after the fusion process.

It can be seen from Tables 1 and 2 that all fusion methods yield high scores for the DHRMIs and LHRMIs. In general, the proposed method produces less spectral distortion than other fusion methods. Hence, the proposed method allows a higher transformation of the texture information of the HRPI when preserving the spectral content of the LRMIs. The proposed method outperforms other fusion methods in fusing the LRMI with the HRPI, because the fusion model takes into account detail injection, as is the case of the EMD based fuser, and spectral signature, as is the case of the GIM based on the SRFs of the sensors. These aspects of the proposed method allow producing the HRMIs closer to the real HRMIs that the QuickBird multispectral sensor would take at the spatial resolution of the HRPI than other fusion methods.

In order to estimate the spatial quality of the HRMIs, we follow the procedure proposed by Zhou [15]. First, the spatial detail information present in the two images to be compared is extracted using the following Laplacian filter. Second, spatial correlation coefficient (SCC) between these two filtered images is calculated. The SCC indicates that how much the detail information of one of the images is present in the other. A high SCC shows that most spatial information of the HPRI has been incorporated into the LRMI during the fusion process:

$$\left|\begin{array}{ccc} -1 & -1 & -1\\ -1 & 8 & -1\\ -1 & -1 & -1 \end{array}\right|$$

Because fusion method injects different amount of details into different band of the LRMIs, for the purpose of evaluating roundly the detail injection performance of fusion method, the average SCC (SCC_avg_) is used as a global spatial quality index for the HRMIs. A good fusion method must allow the injection into each band of the LRMIs of the details the multispectral sensor would capture if it worked at a spatial resolution similar to that of the panchromatic sensor. That means the higher the SCC_avg_ value the higher the spatial quality of the HRMIs. Table 3 shows the results.

The proposed method outperforms the AWT, BT, DWT, HPF, and HPM fusion methods in incorporating spatial details of the HRPI into the LRMIs by taking into account the separation of spatial information from the spectral information, as is the case of the EMD decomposition though the IHS method is the best. This injection model allows producing the HRMIs closer to the real HRMIs that the multispectral sensor would take at the spatial resolution of the HRPI. Visual inspection and quantitative comparison show that the proposed method gets the advantage of many traditional methods in fusing the LRMIs with the HRPI when the HRMIs are compared with the LRMIs.

## 4. Conclusions

In this paper, we wed the ideas of SRF based GIM and the EMD for fusing the LRMI with the HRPI of the same scene in order to obtain one HRMI. The LRIC used in the GIM is obtained from weighted averaging the LRMIs based on the SRFs of the multispectral and panchromatic sensors for separating the low spatial intensity from the spectral information while the EMD is introduced for alleviating the spectral distortion caused by the IHS approach. The LRIC is replaced with the produced HRIC. Finally, the HRMIs are produced by performing the GIM.

QuickBird LRMIs and HRPI are used to demonstrate the advantage of the proposed method over the traditional fusion approaches in terms of preserving the spectral properties of the LRMIs. The experimental results are compared with those of six fusion methods by visual inspection and quantitative comparison. The comparison results confirm the spectral preservation property of the proposed method. All these results are encouraging, and they show that the proposed method can achieve better spectral preservation together with spatial enhancement.

## Figures and Tables

**Table 1.** Values of the six indexes analyzed to evaluate the qualities of DHRMIs

		**IHS**	**AWT**	**BT**	**DWT**	**HPF**	**HPM**	**The proposed method**	**ideal**
CC	B_1_	0.9144	0.9808	0.9649	0.9634	0.9774	0.9765	0.9853	1
	B_2_	0.9177	0.9798	0.9665	0.9689	0.9763	0.9776	0.9867	1
	B_3_	0.9214	0.9797	0.9625	0.9713	0.9762	0.9772	0.9869	1
	B_4_	0.8909	0.9410	0.8011	0.9118	0.9321	0.9353	0.9820	1

RMSE	B_1_	39.451	21.211	25.794	23.635	21.740	19.412	15.313	0
	B_2_	38.134	21.666	26.277	22.263	21.313	18.774	14.452	0
	B_3_	36.265	21.486	27.339	21.336	20.449	18.876	14.314	0
	B_4_	42.942	28.575	55.273	30.160	30.333	29.730	16.757	0

SAM		12.574	6.8855	10.452	8.6793	7.9877	7.8121	5.1365	0

Q_4_		0.8948	0.9615	0.9083	0.9439	0.9562	0.9602	0.9821	1

RASE		28.248	16.986	23.695	17.915	17.540	16.756	11.678	0

ERGAS		5.1954	2.6837	4.4131	3.5957	3.2456	3.2083	2.0846	0

**Table 2.** Values of the six indexes analyzed to evaluate the qualities of LHRMIs

		**IHS**	**AWT**	**BT**	**DWT**	**HPF**	**HPM**	**The proposed method**	**ideal**
CC	B_1_	0.8660	0.9620	0.9588	0.9534	0.9587	0.9610	0.9758	1
	B_2_	0.8669	0.9697	0.9539	0.9545	0.9663	0.9691	0.9754	1
	B_3_	0.8697	0.9622	0.9446	0.9546	0.9607	0.9609	0.9772	1
	B_4_	0.8470	0.9642	.7208	0.9097	0.9561	0.9620	0.9697	1

RMSE	B_1_	45.698	27.537	27.102	26.016	29.216	25.343	25.267	0
	B_2_	45.034	24.862	28.656	25.817	26.745	22.335	21.903	0
	B_3_	44.423	27.137	31.488	26.005	28.064	27.861	20.476	0
	B_4_	46.874	22.007	55.211	41.703	33.649	32.918	21.268	0

SAM		15.593	8.3426	12.588	9.8584	9.4756	8.5456	7.0861	0

Q_4_		0.8487	0.9627	0.8799	0.9398	0.9576	0.9569	0.9673	1

RASE		30.587	16.693	31.492	20.730	23.251	18.181	17.147	0

ERGAS		5.7825	3.0859	5.1949	3.6972	3.7452	3.5916	2.8375	0

**Table 3.** the SCC_avg_ comparison between the spatial detail of the average HRMI and the HRPI

	**IHS**	**AWT**	**BT**	**DWT**	**HPF**	**HPM**	**The proposed method**	**ideal**
SCC_avg_	0.9960	0.9714	0.9505	0.7012	0.9714	0.8688	0.9809	1
